# Letter to the Editor

**DOI:** 10.22037/iej.v12i4.17797

**Published:** 2017

**Authors:** Juan Carlos Valverde Huaranga, Gerson Lara Baldeon, Stefany Caballero-García

**Affiliations:** Universidad Peruana de Ciencias Aplicadas, Peru

Moghadam N, Abdollahi AA, Aghabalayi Fakhim H. *In Vitro* Sealing Properties of Calcium-Enriched Mixture and Mineral Trioxide Aggregate Orifice Barriers during Intra-Coronal Bleaching. **Iran Endod J. 2017;12(2):231-5. *Doi*: 10.22037/iej.2017.45**

We have read with great interest the article by Moghadam *et al*. [1] and we found the study interesting because the authors appropriately dealt with the sealing properties of the Calcium Enriched Mixture and Mineral Trioxide Aggregate used as cervical barriers during the treatment of tooth whitening. It is known that the filtration of hydrogen peroxide in the periodontal space can cause root resorptions at the cervical level [1], therefore it is important to have performed the evaluation in question. However, we would like to comment on a few methodological aspects wish might benefit futures related studies.

Currently there is controversy regarding the measurement of filtration. Although no consensus has been reached on the most appropriate technique for the evaluation of this filtering process, it is known that some methods such as bacterial penetration, fluid filtration and glucose penetration model presents more real results and have less disadvantages for measurement.

Various investigations indicate that the penetration of dyes presents inconveniences by a possible effect of entrained air at the entrance of the dye solution. Previous studies have shown that at normal atmospheric pressure, the effect of air trapped along the margin of the root canal can impede the flow of penetration [2, 3]. Thus, a high degree of variation has been found in the results of penetration, when the same materials and methods were used with different atmospheric pressures [4]. In order to solve this effect, it is recommended to follow the vacuum methodology, in which a partial pressure is applied which facilitates the penetration of the coloring agent [2, 4].

Furthermore, to perform measurements of dye penetration in millimeters, the teeth were cut longitudinally using diamond discs 0.1 mm in diameter. This procedures present also disadvantages, because it is possible that part of the sample is eliminated and consequently, part of the measurement can be lost. A procedure that could prevent these drawbacks is the diaphanization. This technique allows to obtain a three-dimensional view of the root canals. In addition, in comparison to the conventional process of dye penetration measurement, the diaphanization has the advantage of not requiring cutting and practice to perform an evaluation in the three dimensions of the tooth [5].

We hope these recommendations will contribute to enrich future related studies and to better understand the methods used to assess the microleakage of different materials. 
