# Supplementary material for: Letter to the Editor
Source: Iran Endod J. 2017 Fall;12(4):540–1. doi: 10.22037/iej.v12i4.17797 (PMC5722102; doi:10.22037/iej.v12i4.17797)
Supplement: Related texts

# Authors Reply

**Published:** 2017

**Authors:** Amir Ardalan Abdollahi

**Affiliations:** Dental School, West Azarbaijan, Urmia University of Medical Science, Urmia, Iran. ***E-mail***: ardalan_2000a@yahoo.com

Thanks for reading and commenting on our manuscript. Here are the comments and some responses to them:

***1.*** Currently there is controversy regarding the measurement of filtration. Although no consensus has been reached on the most appropriate technique for the evaluation of this filtering process, it is known that some methods such as bacterial penetration, fluid filtration and glucose penetration model presents more real results and have less disadvantages for measurement.

***Response:***

We agree with your comment; however we chose dye penetration in this study since it is a simple and useful method based on chemical color reaction and it has been used in several studies [1, 2]. Also as it was mentioned there has been no agreement about the standard technique for evaluation of sealing properties of materials. Furthermore, other methods have some disadvantages, for example, special species or a limited number of bacteria are used in bacterial leakage tests, therefore it doesn’t provide the real clinical condition. Moreover, bacterial synergistic effect, influence of environment, thermal changes, salivary enzymes, buffering materials and antibodies are neglected in bacterial leakage studies [3]. 

***2.*** Various investigations indicate that the penetration of dyes presents inconveniences by a possible effect of entrained air at the entrance of the dye solution. Previous studies have shown that, at normal atmospheric pressure, the effect of air trapped along the margin of the root canal can impede the flow of penetration. Thus, a high degree of variation has been found in the results of penetration, when the same materials and methods were used with different atmospheric pressures. In order to solve this effect, it is recommended to follow the vacuum methodology, in which a partial pressure is applied which facilitates the penetration of the coloring agent.

***Response***:

It is true that the air trapped along the margin of root canal can impede the flow of penetration, but there is no consensus about the atmospheric pressure in which the dye penetration test should be done. Also no studies have been recommended the standard conditions such as atmospheric pressure to do the dye penetration test.

***3.*** Furthermore, to perform measurements of dye penetration in millimeters, the teeth were cut longitudinally using diamond discs of 0.1 mm in diameter. This procedures present also disadvantages, because it is possible that part of the sample is eliminated and consequently, part of the measurement can be lost. A procedure that could prevent these drawbacks is the diaphanization. This technique allows to obtain a three-dimensional view of the root canals. In addition, in comparison to the conventional process of dye penetration measurement, the diaphanization has as main advantage that it does not require cutting and practice to perform an evaluation in the three dimensions of the tooth.

***Response:***

We admire your recommendation; however there were no facilities of diaphanization in our faculty or other Iranian Dental Faculties. Also diaphanization has not been a common method and no previous studies have been used this procedure and cutting the teeth with discs has been used.

## References

[1] Brito-Junior M, Faria-e-Silva AL, Fonseca B, Camilo CC (2009). Sealing ability of MTA used as cervical barrier in intracoronal bleaching. Acta Odontol Latinoam.

[2] Beckham BM, Anderson RW, Morris CF (1993). An evaluation of three materials as barriers to coronal microleakage in endodontically treated teeth. J Endod.

[3] Moradi S, Disfani R, Ghazvini K, Lomee M (2013). Sealing Ability of Orthograde MTA and CEM Cement in Apically Resected Roots Using Bacterial Leakage Method. Iran Endod J.
